# Differences in Root-Zone Soil and Basal Stem Tissue Microbial Communities Between Asymptomatic and Diseased *Citrus maxima* var. Tomentosa Plants with Foot Rot

**DOI:** 10.3390/microorganisms14102258

**Published:** 2026-10-04

**Authors:** Zongqin Feng, Yin Liu, Haijia Lin, Fengcui Zhou, Zhidong Zheng, Qinqin He, Xinmin Huang

**Affiliations:** Guangdong Provincial Key Laboratory of Green and Intelligent Agricultural Production, College of Biological and Food Engineering, Guangdong University of Petrochemical Technology, Maoming 525000, China; zongqinfeng@163.com (Z.F.); liu.yin@gdupt.edu.cn (Y.L.); haijia345929@163.com (H.L.); z13664904935@163.com (F.Z.); zzd060616@163.com (Z.Z.); xiaohe1507@126.com (Q.H.)

**Keywords:** soil-borne disease, bacterial community, fungal community, *F. solani-melongenae*, Koch’s postulates

## Abstract

*Exocarpium Citri grandis*, derived from *Citrus maxima* var. Tomentosa, is a precious traditional Chinese medicinal herb. However, foot rot, a severe soil-borne disease, threatens the sustainable cultivation of this crop. This exploratory study investigated microbial associations with foot rot using a limited sample set from a single orchard. Microbial communities in root-zone soil and basal stem tissues of asymptomatic and foot-rot-infected trees were analyzed via high-throughput sequencing. Significant differences in microbial diversity and community structure were observed between asymptomatic and foot-rot-infected trees. Root-zone microbial diversity was lower in diseased trees, whereas bacterial diversity increased but fungal diversity decreased in diseased basal stem tissues. Linear discriminant analysis effect size and correlation analyses revealed that beneficial prokaryotic genera, such as *Bacillus*, were negatively correlated with *Fusarium*. FUNGuild prediction indicated a functional shift in fungal communities from saprotrophic to pathogenic. *Fusarium* was significantly enriched in diseased samples; the HZY isolate from diseased tissues was identified as *Fusarium solani-melongenae*. Pathogenicity assays demonstrated that *F. solani-melongenae* was capable of causing foot rot symptoms. These hypothesis-generating findings suggest microbial associations that may underlie foot rot pathogenesis and provide a basis for formulating testable hypotheses for sustainable disease management strategies, pending validation in larger, multi-site studies.

## 1. Introduction

*Exocarpium Citri grandis* (ECG), from *Citrus maxima* var. Tomentosa, is a traditional medicinal herb endemic to China. This herb exhibits multiple pharmacological properties, including antitussive, expectorant, anti-inflammatory, and antioxidant activities, as well as lung-moistening and anti-asthmatic effects [1,2,3]. Moreover, it is widely used to treat cough, chronic bronchitis, pharyngitis, dyspeptic abdominal distension, and other disorders [4,5]. Modern pharmacological studies have confirmed that ECG can alleviate nonalcoholic fatty liver disease, hyperlipidemia, and diabetes mellitus [6,7,8].

*Citrus maxima* var. Tomentosa is cultivated in Huazhou, Guangdong Province, where it is the dominant economic crop. The cultivation scale and fruit quality of this variety directly determine the yield and medicinal efficacy of ECG. This crop plays a key role in the local economy, and its conservation is important for maintaining medicinal germplasm resources and establishing standardized cultivation systems for traditional Chinese medicinal materials [9]. Recently, large-scale continuous monocultures and longer cultivation periods have increased the incidence of soil-borne diseases in *C. maxima* var. Tomentosa orchards [9]. Among the diseases, foot rot, a soil-borne disease, exhibits an annual tendency to spread. This disease limits the sustainable development of the *C. maxima* var. Tomentosa industry [10], as it is one of the most destructive citrus diseases. It mainly infects the basal stem (the junction of the stem and root-zone soil) and root tissues of host plants. Foot rot typically begins when pathogens invade host tissues through wounds or natural cavities, resulting in cortical rot of the stem base, vascular bundle necrosis, and gummosis of trunks and branches. With disease progression, these symptoms lead to leaf chlorosis, decreased tree vigor, whole-plant wilting, and death under severe infection [11,12].

Preliminary field surveys have revealed that foot rot disease in this medicinal pomelo initially emerged in Pingding Town in 2019 and subsequently spread on a large scale in 2023. This disease substantially reduces the yield and medicinal quality of *C. maxima* var. Tomentosa, causes premature tree senescence, and shortens the orchard renewal cycle, resulting in considerable economic losses to local growers. Disease management relies on chemical pesticide application and soil disinfection; however, these strategies frequently lead to pesticide residue pollution, disruption of soil microecological balance, and the emergence of fungicide resistance in pathogens [13]. Therefore, identifying the causal pathogen and understanding its pathogenic mechanisms are essential for establishing green, efficient, and sustainable integrated disease management systems. Notably, citrus foot rot is mainly caused by *Phytophthora parasitica* Dastur, *P. citrophthora* (R.E. Sm. & E.H. Sm.) Leonian, *P. capsici* Leonian, and other species within the genus *Phytophthora* [11,12,14,15]. In addition, pathogens such as *Microdochium nivale* (Fr.) Samuels and Hallett, *Fusarium* spp., and *Diaporthe destruens* (Harter) Hirooka, Minosh. & Rossman can cause foot rot in other plant species [16,17,18,19]. However, the comprehensive pathogenesis of foot rot in *C. maxima* Tomentosa remains poorly understood, and the complex composition of the pathogenic microorganisms poses considerable challenges for efficient disease management.

Plant roots, root exudates, soil substances, and microorganisms interact synergistically to form a distinctive and dynamic rhizosphere microenvironment [20,21,22]. Through long-term co-evolution, plants and root-zone microorganisms establish a stable symbiotic microecosystem through diverse interactions, including symbiosis, antagonism, parasitism, and mutualism. These interactions occur between host plants and microorganisms, directly modulating plant growth, development, stress, and disease resistance [20,23,24]. In healthy rhizosphere ecosystems, balanced niche competition among microbial taxa can effectively suppress massive colonization by pathogenic microbes. In contrast, deteriorated soil conditions and disrupted microbial community structure disrupt this competitive balance, creating vacant ecological niches that facilitate the invasion, colonization, and spread of soil-borne pathogens, ultimately leading to plant disease occurrence [24,25]. Healthy and diseased plants exhibit significant differences in the composition, diversity, and species abundance of root-zone microbial communities [26,27,28,29]. For example, in *Rosa roxburghii* Tratt, root rot disease markedly alters the diversity, structure, and composition of rhizosphere microbial communities, with pathogenic fungi accumulating gradually over time [26]. Compared with healthy *Zanthoxylum armatum* DC. trees, those infected with root rot exhibit decreased richness of rhizospheric bacterial and fungal communities, accompanied by a substantial increase in the relative abundance of *Fusarium* spp. [27]. In continuously cropped *Atractylodes macrocephala* Koidz., rhizosphere microecological imbalance, driven by shifts in soil microbial communities, is the core cause of the obstacles to continuous cropping [28]. Furthermore, a comparative analysis of the physicochemical properties and microbial diversity between asymptomatic and diseased *A. macrocephala* has confirmed that *Fusarium* was the predominant pathogen responsible for root rot, while disease was associated with considerable reductions in the diversity and richness of endophytic bacteria and fungi in the host plants [29].

However, most studies on citrus foot rot have mainly focused on common citrus cultivars and the Shatian pomelo. Although *Phytophthora* has been identified as a dominant pathogen, most studies have focused on pathogen isolation and identification, with a few having analyzed both root-zone soil and endophytic microorganisms within host tissues. For *C. maxima* var. Tomentosa, previous research has predominantly focused on cultivation techniques, identification of active ingredients, and evaluation of germplasm resources. However, the pathogenic species and characteristics of foot rot in this plant remain underexplored, and the differences in microbial communities in the root-zone and basal stem tissues between asymptomatic and diseased trees have not been systematically investigated.

In view of the above research gaps, root-zone soil and basal stem tissues collected from asymptomatic and typical foot-rot-infected *C. maxima* var. Tomentosa plants were selected as research materials for the present study. High-throughput sequencing targeting the fungal internal transcribed spacer (ITS) region and the bacterial 16S rRNA gene was performed to analyze differences in the structure and diversity across samples. Based on pathogen isolation and purification, morphological observations, molecular identification, and experimental inoculation assays, the identity of the primary pathogenic fungi causing foot rot in *C. maxima* var. Tomentosa were confirmed. This study aimed to elucidate the microecological mechanisms underlying foot rot outbreaks and to provide a theoretical basis for orchard soil improvement, regulation of root-zone microecology, targeted disease control, and eco-friendly, high-quality, standardized cultivation of this geo-authentic medicinal herb.

## 2. Materials and Methods

### 2.1. Sample Collection

Samples were collected from Pingding Town, Huazhou City, Guangdong Province, China, which is a core production region of *C. maxima* var. Tomentosa. On 21 June 2023, soil and stem tissue samples were collected from a commercial plantation in Pingding Town (110°25′33.33″ E, 22°0′45.41″ N). The site contained 12–15-year-old air-layered trees of *C. maxima* var. Tomentosa (‘Zhengmao’). Notably, uniform agronomic management practices, including fertilization, irrigation, and disease control regimes, have been implemented throughout the plantation, which minimized the confounding impact of tree-to-tree variability on the observed microbial patterns.

**Figure 1:**
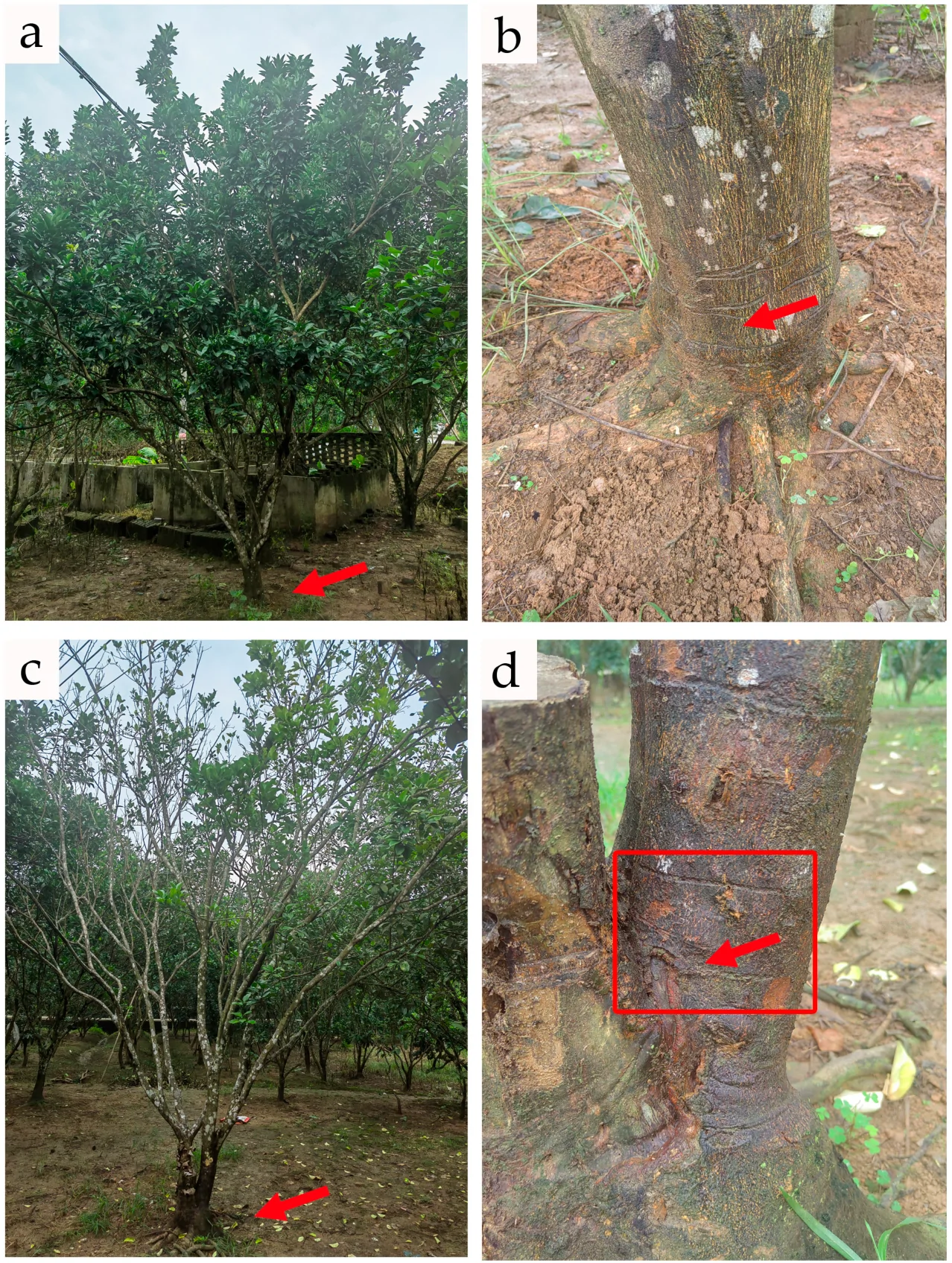
Morphological traits and sampling sites of asymptomatic and foot rot-infected *Citrus maxima* var. Tomentosa trees. (**a**) Morphology of asymptomatic tree; (**b**) sampling site of asymptomatic tree; (**c**) morphology of diseased tree; (**d**) sampling site of diseased tree. Red arrows indicate the sampling positions, and the red box marks the diseased basal stem tissue.

A case-control sampling strategy was applied within a 1333.3 m^2^ homogeneous plot in the orchard. Based on a field survey of stem-base symptoms, the disease incidence was estimated at 19%, with symptomatic trees identified morphologically. Basal stem tissues and root-zone soil were collected from four trees with foot rot symptoms and four asymptomatic trees. Each selected diseased tree was separated from the selected asymptomatic trees by at least four intervening asymptomatic trees to avoid spatial autocorrelation. A total of 16 samples were obtained: basal stem tissues from asymptomatic trees were coded as NT1–NT4, with their corresponding root-zone soil samples labeled as NS1–NS4; basal stem tissues from diseased trees were coded as DT1–DT4, with their corresponding root-zone soil samples labeled as DS1–DS4 (Figure 1).

Root-zone soil samples were collected from a circular zone (0.5 m in diameter) centered on the tree trunk. The soil collected by this procedure is referred to as ‘root-zone soil’. Before sampling, surface litter, fallen leaves, and debris were removed. Soil samples were excavated using a sterile stainless-steel shovel and immediately transferred into 50 mL sterile centrifuge tubes. For diseased trees, the visibly necrotic, water-soaked, or decaying outer layers were carefully removed and discarded from the basal stem (15–25 cm above ground); only symptomatic but structurally intact basal stem tissue—i.e., discolored yet firm, non-decaying tissue at the margin of the lesion—was collected. Symptomatic tissue blocks were excised, surface-sterilized by immersion in 75% (*v*/*v*) ethanol for 1 min, washed with sterile distilled water four or five times, and placed into sterile 50 mL centrifuge tubes using sterile forceps. For asymptomatic trees, intact epidermal tissues were harvested at the same stem height and processed similarly. All instruments and consumables used throughout the sampling procedure were sterile. To prevent cross-contamination, a new set of sterile tools, including sterile gloves, was used for each sample. All samples were snap-frozen in liquid nitrogen immediately after collection and transported on dry ice to the laboratory for subsequent microbial community profiling.

### 2.2. DNA Extraction, Polymerase Chain Reaction (PCR) Amplification, Sequencing, and Data Processing

Total microbial genomic DNA was extracted from 0.5 g (fresh weight) of each root-zone soil sample and 200 mg (fresh weight) of each basal stem tissue sample using the FastDNA^®^ Spin Kit (MP Biomedicals, Irvine, CA, USA) [30]. The quality and concentration of extracted DNA were assessed using 1.0% agarose gel electrophoresis and a NanoDrop^®^ ND-2000 spectrophotometer (Thermo Scientific, Waltham, MA, USA), respectively. Target fragments were amplified via PCR using Q5^®^ High-Fidelity DNA Polymerase (New England Biolabs, Euroclone, Milan, Italy). The fungal internal transcribed spacer *(ITS)1* region was amplified using the *ITS1F* and *ITS2* primers [31]. The V3–V4 hypervariable region of the bacterial 16S rRNA gene was amplified using primers 341F and 806R [32]. A negative control, in which nuclease-free water was substituted for the template, was included in every PCR run to monitor reagent contamination. Amplicons were separated by 2% agarose gel electrophoresis, and only those assays in which no band was detected in the negative control were considered valid. All qualified PCR products were purified with AMPure XP beads (Beckman Coulter, High Wycombe, UK) to remove nonspecific fragments and primer dimers. The concentration of purified amplicons was accurately quantified using a Qubit 3.0 Fluorometer (Life Technologies, Carlsbad, CA, USA). Sequencing libraries were constructed using the Illumina DNA Prep Kit (Illumina, San Diego, CA, USA), according to the manufacturer’s protocols. Library quality and concentration were validated using the ABI StepOnePlus Real-Time PCR System (Applied Biosystems, Waltham, MA, USA). Qualified libraries were pooled in equal amounts and sequenced on the Illumina NovaSeq 6000 (Illumina) platform in the PE250 paired-end sequencing mode. All raw sequence data have been deposited at the National Genomics Data Center (https://ngdc.cncb.ac.cn/) under the accession number PRJCA064203.

### 2.3. Microbiome Bioinformatics Analysis

Reads with ≥10% unknown nucleotides (N), reads containing adapters, and low-quality reads were removed using FASTP (version 0.18.0). The quality-filtered paired reads were then assembled into Tags using FLASH (version 1.2.11) with a minimum overlap length of 10 bp and a maximum mismatch rate of 2%. The resulting Raw Tags were further stringently filtered to obtain high-quality Clean Tags. Clean Tags were clustered into Operational Taxonomic Units (OTUs) at a 97% similarity threshold using the UPARSE algorithm implemented in USEARCH (version 11.0.667) [33], and the most abundant sequence within each OTU was selected as the representative sequence. The 97% sequence-similarity clustering threshold follows the conventional species-level operational definition that is widely adopted in plant microbiome studies, and it adequately supports community-level structural characterization as well as the identification of genus-level differentially abundant taxa and co-occurrence associations [33]. Chimeras were removed using the UCHIME algorithm, and the filtered Effective Tags were used for OTU abundance statistics and downstream analyses. To avoid bias introduced by unequal sequencing depth, all samples were rarefied to the minimum number of effective tags across the dataset, and all downstream diversity and community analyses were based on the rarefied OTU tables. OTU representative sequences were aligned against the corresponding reference databases: SILVA (version 138.2) for 16S data and UNITE (version 7.2) for ITS data. Taxonomic annotation was performed using the RDP classifier (version 2.14) with a confidence threshold of 80% [34]. Because plant stem tissues were analyzed, sequences annotated as chloroplasts, mitochondria, or unclassified at the kingdom level were removed in subsequent analyses.

The Chao1, ACE, Shannon, and Simpson diversity indices were calculated in R (version 4.3.1), and the rarefaction curves and rank–abundance curves were plotted using the ggplot2 package (version 4.0.1). Weighted and unweighted UniFrac distance matrices were computed from the OTU sequences using the GUniFrac package (version 1.8). Based on the abundance table, the vegan package (version 2.5.6) was used to calculate Bray–Curtis, Jaccard, unweighted UniFrac, and weighted UniFrac distance matrices. Principal Coordinates Analysis (PCoA) was subsequently performed based on Bray–Curtis distances to evaluate differences in community composition among samples. Fungal functional guilds were predicted using FUNGild database [35]. The correlations among the top 20 most abundant fungal and bacterial genera were analyzed using the Sparse Correlations for Compositional Data (SparCC) algorithm implemented in the R package SpiecEasi (version 1.1.0). Edges with |r| > 0.5 and *p* < 0.05 were selected, and the resulting genus-level association network was visualized on the OmicShare platform (https://www.omicshare.com) [36].

### 2.4. Isolation of Microorganisms from Diseased Basal Stem Tissues of Citrus maxima var. Tomentosa

Micoorganisms were isolated using diseased basal stem tissues from the same four trees specified in Section 2.1. The tissues were excised with a sterile blade, immediately sealed in sterile plastic bags, transported to the laboratory at 4 °C, and processed for pathogen isolation within 24 h. The diseased tissues were rinsed with sterile distilled water three times, surface-sterilized via immersion in 75% (*v*/*v*) ethanol for 30 s, and washed four or five times with sterile distilled water. Under aseptic conditions, the sterilized tissues were cut into 5 mm × 5 mm pieces and plated onto potato dextrose agar (PDA, Huankai, Guangzhou, China). Cultures were incubated at 28 °C in the dark for 7 days. For strain purification, hyphae from colony margins were picked and subcultured onto fresh PDA medium.

The mycelial and conidial morphological characteristics of purified isolates were observed under an optical microscope (BMC500, Phenix, Shangrao, China). Fungal genomic DNA was directly extracted from fresh mycelia using a lysis buffer for PCR, and the supernatant was used as the PCR template after brief centrifugation. PCR amplification was performed using the universal fungal primer sets ITS1/ITS4 [37], EF-1/EF-2 [38], and fRPB2-5f2/fRPB2-7cR [39]. Purified PCR products were sent to Tianyi Huiyuan Biotechnology (Guangzhou, China) for Sanger sequencing. The obtained sequences were BLASTed (version 2.17.0) against the NCBI database, and phylogenetic trees were constructed in MEGA 12 [40] using the neighbor-joining (NJ) method with the Kimura 2-parameter (K2P) model, pairwise deletion, and 1000 bootstrap replicates; the tree was rooted with Fusarium oxysporum species complex (FOSC) as the outgroup.

### 2.5. Pathogenicity Assay and Pathogen Re-Isolation

The pathogenicity of the isolates was evaluated according to Koch’s postulates. One-year-old healthy potted *C. maxima* var. Tomentosa (‘Zhengmao’) seedlings and excised current-year branches were used as host materials. Before inoculation, the basal stem surface of seedlings was disinfected with 75% (*v*/*v*) ethanol, and uniform wounds were created using a sterile scalpel. The previously isolated and identified pathogenic fungus was inoculated onto PDA plates. Following 4 days of incubation, mycelial plugs (0.5 cm in diameter) were prepared with a sterile cork borer, and each plug was affixed to the wound site of test trees with the mycelial side closely attached to the injured tissue. The inoculation sites were covered with sterilized moist absorbent cotton and wrapped with sterile parafilm to maintain moisture. Sterile PDA plugs without mycelia were set as the blank control.

Each treatment included three biological replicates (three seedlings), with one inoculation site per seedling. All seedlings were incubated in a plant growth chamber at 28 °C under a 12-h light/dark photoperiod and 75% relative humidity. Disease development was observed at 21 days post-inoculation (dpi). For excised current-year branches, pathogen inoculation was performed following the same procedure applied to seedlings. Inoculated branches were maintained in a climate chamber at 28 °C with a 12-h light/dark photoperiod and 75% relative humidity, and disease symptoms were examined at 10 dpi. To fulfill Koch’s postulates, the pathogen was re-isolated from symptomatic tissues of all inoculated seedlings and branches and subsequently identified through morphological observation and molecular biological assays.

### 2.6. Statistical Analysis

All statistical analyses were performed using R (version 4.3.1) and the Omicsmart online analysis platform (http://www.omicsmart.com). Differences in alpha diversity indices and taxonomic abundances between groups were assessed using the Wilcoxon rank-sum test, with *p* < 0.05 considered statistically significant; because each alpha-diversity index was compared between the two groups in a single hypothesis test, no multiple-testing correction (e.g., FDR) was required. LEfSe (version 1.0) was used to identify bacterial taxa with significantly different abundances among groups from the phylum to genus levels, using the criteria LDA > 2 and *p* < 0.05 [41]. Based on Bray–Curtis distances, permutational multivariate analysis of variance (PERMANOVA; Adonis) with 999 permutations was used to assess the significance of between-group differences in community structure, whereas PERMDISP with 999 permutations was used to assess within-group homogeneity. For cross-kingdom correlations between fungal and bacterial genera, the association network was constructed as an exploratory, hypothesis-generating analysis using the dual threshold |r| > 0.5 and *p* < 0.05. Because this network analysis is exploratory and hypothesis-generating rather than a confirmatory test of pre-specified hypotheses, multiple-testing correction (FDR/Benjamini–Hochberg) was not applied to edge selection.

## 3. Results

### 3.1. Quality Evaluation of High-Throughput Sequencing Data

**Figure 2:**
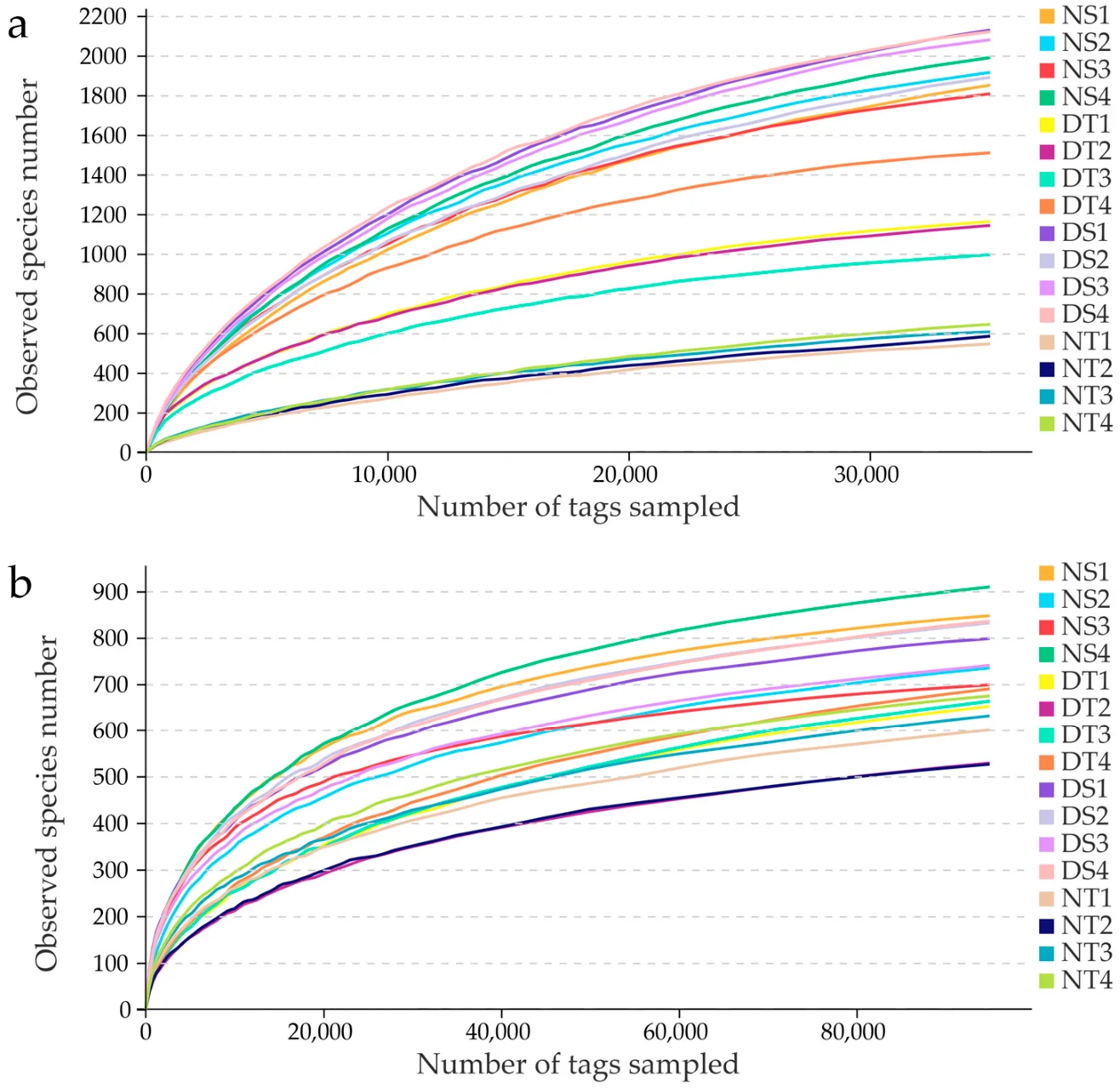
Rarefaction curves of microbial sequencing depth in root-zone soil and stem base. (**a**) Bacteria; (**b**) fungi. NS: asymptomatic tree root-zone soil; NT: Asymptomatic-tree basal stem tissue; DS: diseased-tree root-zone soil; DT: diseased-tree basal stem tissue.

High-throughput sequencing was performed to characterize the microbial communities in the basal stem tissues and root-zone soil collected from asymptomatic and foot-rot-infected *C. maxima* var. Tomentosa trees. For bacterial 16S rRNA gene sequencing, 2,081,668 raw reads were obtained from 16 samples (mean 130,104 per sample; range 121,381–137,862) (Table S1). After strict quality filtering, 1,686,416 Effective Tags were retained (mean 105,401 per sample; range 97,940–113,684), with read lengths ranging from 201 to 474 bp, and 11,205 bacterial OTUs were clustered at a 97% similarity threshold (Table S1). A total of 2,057,477 raw fungal ITS sequences were generated (mean 128,592 per sample; range 121,399–137,452), and 1,747,689 Effective Tags were obtained after filtration (mean 109,231 per sample; range 98,197–119,712), with sequence lengths between 201 and 474 bp, corresponding to 2267 fungal OTUs at the same 97% threshold (Table S2). To equalize sequencing depth across samples, all samples were rarefied to the minimum effective read count (97,940 reads for bacteria and 98,197 reads for fungi). Rarefaction curve analysis at 97% sequence similarity revealed that all curves gradually reached saturation, confirming that the sequencing depth was sufficient to comprehensively assess the diversity of the bacterial and fungal communities in all samples (Figure 2).

### 3.2. Microbial Community Diversity in Basal Stem Tissues and Root-Zone Soil

**Figure 3:**
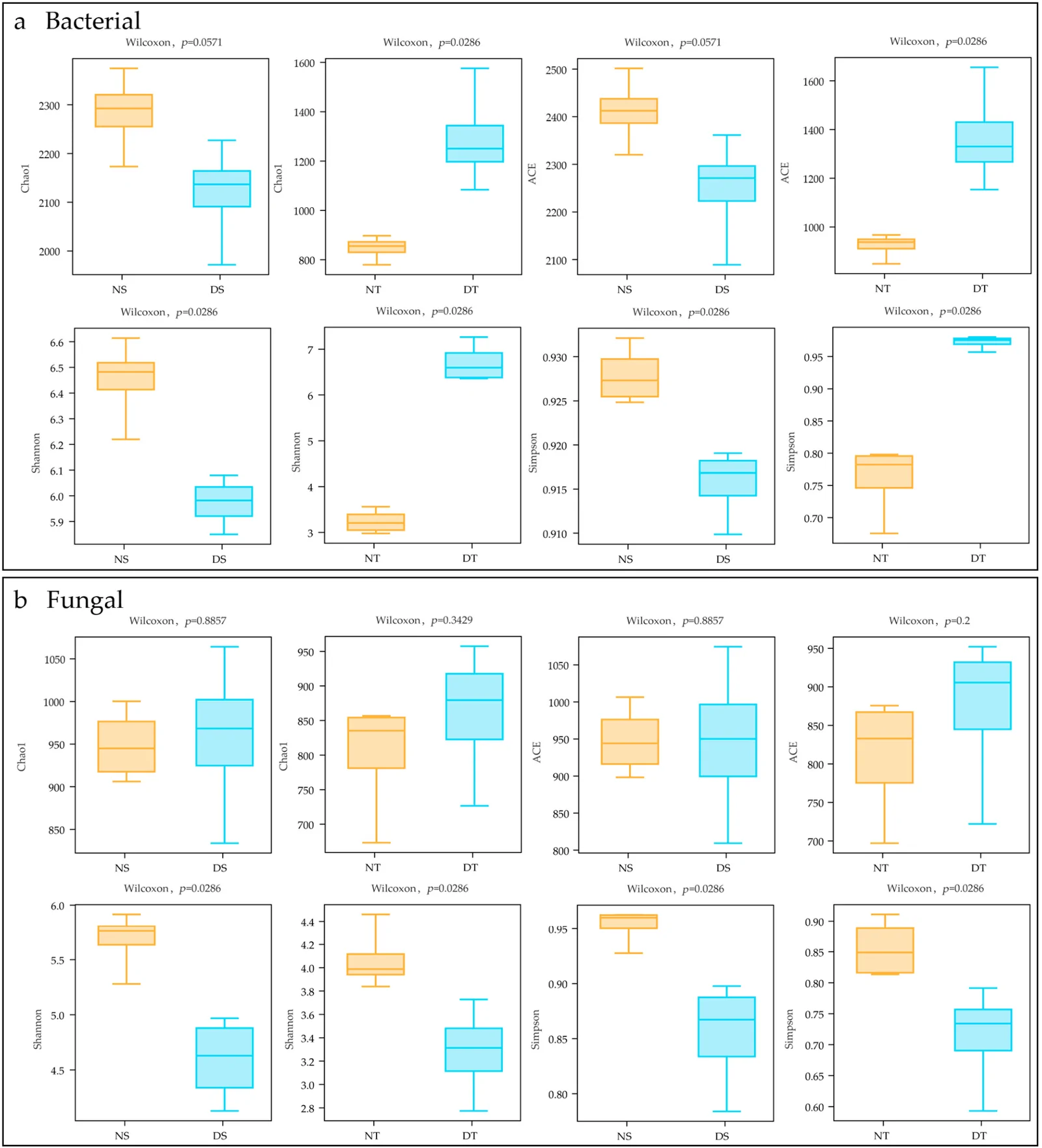
Comparison of the alpha diversity indices of bacterial (**a**) and fungal (**b**) communities in the root-zone soil (NS/DS) and basal stem tissues (NT/DT) of asymptomatic and diseased plants. Box plots show the median (line inside the box), interquartile range (box), and data range (whiskers). NS: root-zone soil from asymptomatic trees; NT: basal stem tissue from asymptomatic trees; DS: root-zone soil from diseased trees; DT: basal stem tissue from diseased trees. Differences between groups were assessed using the Wilcoxon rank-sum test, and the *p*-values are indicated above each subplot; *n* = 4 per group.

The number of bacterial OTUs in the root-zone soil of diseased trees was slightly lower than that in asymptomatic trees, whereas diseased basal stem tissues contained more bacterial OTUs than did asymptomatic ones (Table S1). Alpha diversity analysis showed that, in the root-zone soil, the Shannon and Simpson indices of diseased plants (DS) were significantly lower than those of asymptomatic plants (NS), whereas the Chao1 and ACE richness indices showed a decreasing trend that did not reach statistical significance, indicating an association between foot rot and reduced bacterial alpha diversity in the root-zone microenvironment (Figure 3a). In contrast, in the stem base tissues, the Chao1, ACE, Shannon, and Simpson indices of diseased plants (DT) were all significantly higher than those of asymptomatic plants (NT) (Figure 3a), indicating that the occurrence of foot rot was accompanied by elevated bacterial alpha diversity in symptomatic host tissues.

**Figure 4:**
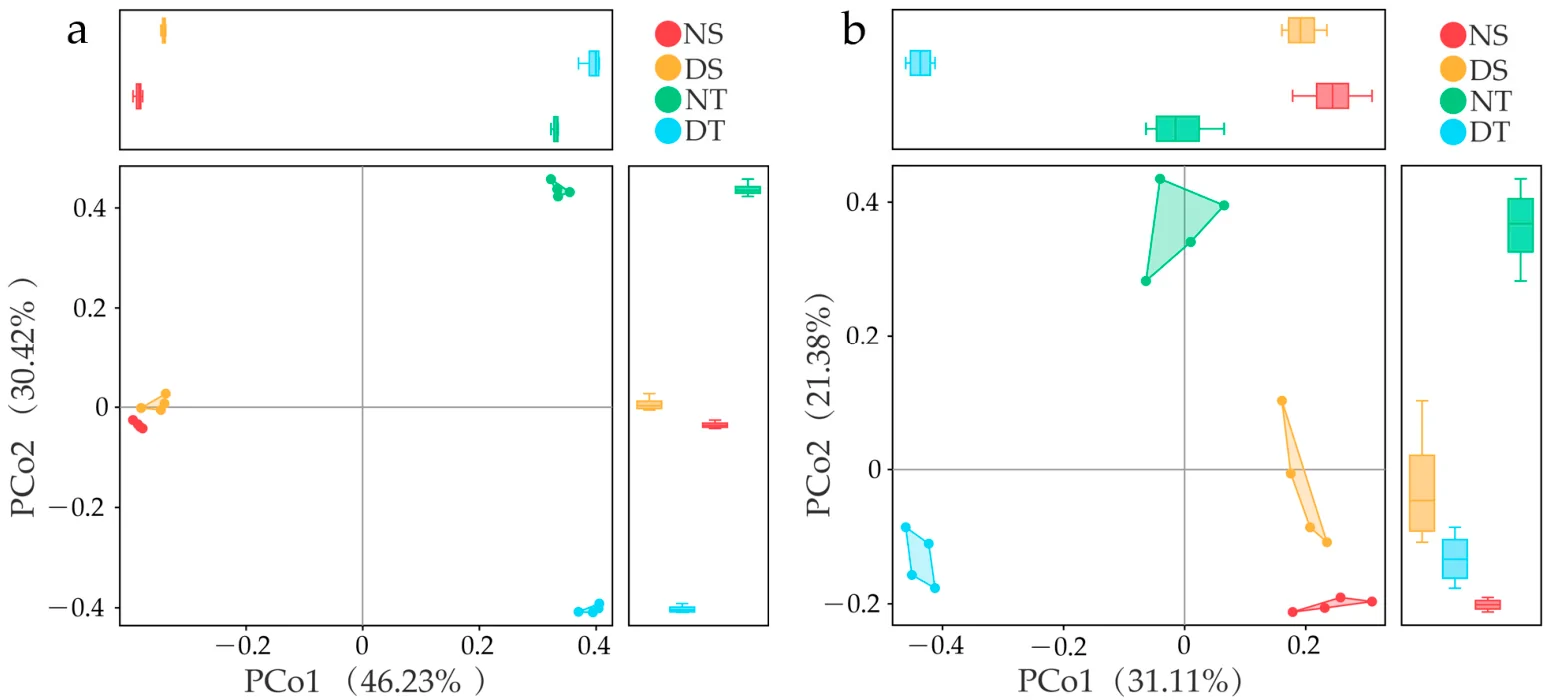
Principal coordinate analysis (PCoA) of microbial communities in root-zone soil and stem basal tissues of asymptomatic and diseased trees. PCoA was performed based on the Bray–Curtis distance algorithm to illustrate the β-diversity among different samples. (**a**) Bacteria; (**b**) fungi. NS: Asymptomatic-tree root-zone soil; NT: Asymptomatic-tree basal stem tissue; DS: diseased-tree root-zone soil; DT: diseased-tree basal stem tissue.

Beta diversity of the bacterial communities differed significantly between asymptomatic (NS, NT) and diseased (DS, DT) plants. PCoA based on Bray–Curtis distances showed a clear separation of asymptomatic samples (NS, NT) from diseased samples (DS, DT), with the first and second principal coordinates explaining 46.23% and 30.42% of the total variation, respectively (Figure 4a). PERMANOVA (Adonis) further confirmed that the bacterial communities in root-zone soil differed significantly between asymptomatic and diseased plants (F = 4.3241, R^2^ = 0.4188, *p* = 0.031), and this difference was also significant in basal stem tissue (F = 14.1298, R^2^ = 0.7019, *p* = 0.033) (Table 1). Dispersion tests indicated that the bacterial community dispersion in diseased basal stem tissue was significantly higher than that in asymptomatic basal stem tissue (F = 26.266, *p* = 0.030) (Table 2), whereas no significant difference was detected in root-zone soil, indicating greater compositional heterogeneity of the bacterial community in diseased basal stem tissue. Taken together, the root-zone soil bacterial communities of asymptomatic and diseased plants differed significantly in community composition, whereas in basal stem tissue, the differences between asymptomatic and diseased bacterial communities were reflected not only in community composition, but also in the higher within-group heterogeneity of diseased plants.

No evident difference was found in fungal OTU numbers between asymptomatic and diseased root-zone soils, whereas diseased basal stem tissues possessed slightly more fungal OTUs than asymptomatic tissues (Table S2). Significant differences in fungal diversity were observed in both the root-zone soil and basal stem tissues between asymptomatic and diseased trees. Regarding the fungal community, regardless of whether in the root-zone soil or the basal stem tissues, the Chao1 and ACE richness indices did not differ significantly between asymptomatic and diseased groups (Figure 3b). However, the Shannon and Simpson diversity indices were significantly lower in diseased samples: the indices in diseased root-zone soil (DS) were significantly lower than those in asymptomatic root-zone soil (NS), and the indices in diseased basal stem tissues (DT) were significantly lower than those in asymptomatic basal stem tissues (NT) (Figure 3b), indicating that the occurrence of foot rot was associated with reduced fungal alpha diversity in both the root-zone microenvironment and host tissues.

PCoA based on Bray–Curtis distances revealed a clear clustering pattern of the fungal communities, separating asymptomatic samples (NS, NT) from diseased samples (DS, DT), indicating that fungal beta diversity differed between asymptomatic and diseased plants (Figure 4b). PERMANOVA (Adonis) showed that the fungal communities in root-zone soil differed significantly between asymptomatic and diseased plants (F = 3.0485, R^2^ = 0.3369, *p* = 0.024), and a significant difference was also observed between asymptomatic and diseased basal stem tissue (F = 8.4646, R^2^ = 0.5852, *p* = 0.020) (Table 1). Dispersion tests revealed no significant difference in fungal community dispersion between asymptomatic and diseased root-zone soil samples (F = 8.0068, *p* = 0.057), whereas the difference was significant in basal stem tissue (F = 14.1444, *p* = 0.028) (Table 2), indicating that the fungal community in asymptomatic basal stem tissue exhibited greater within-group variation. Thus, the fungal communities in root-zone soil differed significantly between asymptomatic and diseased plants in terms of community composition, whereas in basal stem tissue, the differences encompassed both community composition and within-group heterogeneity.

### 3.3. Microbial Community Structure in Basal Stem Tissues and Root-Zone Soil

#### 3.3.1. Bacterial Community Structure

**Figure 5:**
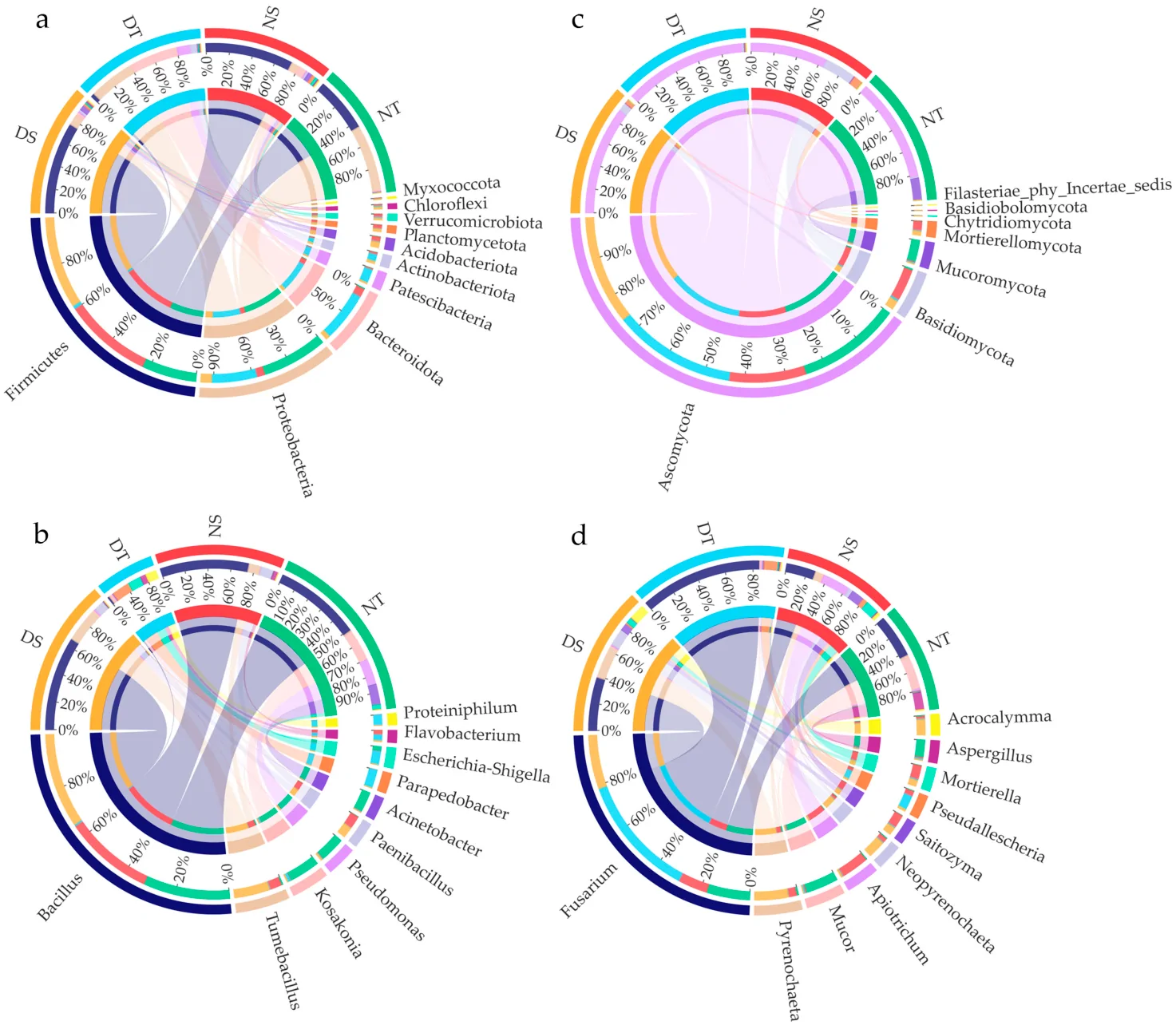
Circos plots depict dominant bacteria and fungi in root-zone soil and stem basal tissues of asymptomatic and diseased trees. (**a**) Phylum-level abundance analysis of bacterial communities; (**b**) genus-level abundance analysis of bacterial communities; (**c**) phylum-level abundance analysis of fungal communities; (**d**) genus-level abundance analysis of fungal communities. NS: Asymptomatic-tree root-zone soil; NT: Asymptomatic-tree basal stem tissue; DS: diseased-tree root-zone soil; DT: diseased-tree basal stem tissue.

Distinct differences in bacterial community composition were observed between asymptomatic and foot rot-infected basal stem tissues and root-zone soil. At the phylum level, Proteobacteria (52.92%) and Firmicutes (44.18%) dominated asymptomatic basal stem tissues, whereas Bacteroidetes (39.57%), Proteobacteria (36.52%), and Patescibacteria (10.90%) were predominant in diseased tissues. In asymptomatic root-zone soil, Firmicutes accounted for 70.88%, followed by Bacteroidetes (6.72%) and Proteobacteria (6.34%). In the diseased root-zone soil, Firmicutes remained the dominant phylum with a relative abundance of 73.31%, and Proteobacteria accounted for 8.49% (Figure 5a).

At the genus level, *Bacillus*, *Kosakonia*, *Pseudomonas*, and *Acinetobacter* were predominant in asymptomatic basal stem tissues, with relative abundances of 42.78%, 15.23%, 12.46%, and 9.95%, respectively. The original dominant bacterial genus pattern was disrupted in diseased tissues, and most bacterial genera exhibited low relative abundance. Genera with relative abundances exceeding 5% included *Parapedobacter* (8.98%), *Escherichia-Shigella* (6.49%), and *Proteiniphilum* (5.14%). In asymptomatic root-zone soil, *Bacillus* was the dominant genus (43.77%), followed by *Paenibacillus* (6.24%) and *Tumebacillus* (5.85%). In diseased root-zone soil, *Bacillus* (45.99%) and *Tumebacillus* (17.38%) were the dominant genera, and *Paenibacillus* accounted for 5.16% (Figure 5b).

#### 3.3.2. Fungal Community Structure

Ascomycota (81.02%) and Mucoromycota (16.99%) were the dominant fungal phyla in asymptomatic basal stem tissues, whereas Ascomycota dominated the diseased tissues with a relative abundance of 96.36%. In asymptomatic root-zone soil, Ascomycota (60.44%) and Basidiomycota (24.87%) were predominant, and Mortierellomycota accounted for 7.06%. In diseased root-zone soil, Ascomycota and Basidiomycota accounted for 84.56% and 6.66%, respectively (Figure 5c).

At the genus level, *Fusarium*, *Mucor*, and *Aspergillus* were dominant in asymptomatic basal stem tissues with proportions of 22.38%, 16.87%, and 8.91%, respectively. *Fusarium* became overwhelmingly dominant in diseased tissues, with a high abundance of 62.97%, whereas *Pseudallescheria* accounted for 7.26%. Fungal genera were evenly distributed in the asymptomatic root-zone soil, and genera with a relative abundance of over 5% included *Fusarium* (15.21%), *Apiotrichum* (14.30%), *Mortierella* (7.00%), and *Saitozyma* (6.74%). The dominant fungal genera in the diseased root-zone soil were *Fusarium* (27.33%), *Pyrenochaeta* (17.24%), *Neopyrenochaeta* (8.93%), and *Acrocalymma* (7.87%) (Figure 5d). Notably, the relative abundance of *Fusarium* significantly increased in both diseased tissues and root-zone soil, whereas that of *Pyrenochaeta* increased in the root-zone soil of the diseased plants.

#### 3.3.3. Differential Analysis of Microorganisms Between Asymptomatic and Diseased Samples

To further explore the effects of foot rot on microbial communities in basal stem tissues and root-zone soil, LEfSe was used to screen differential microbial taxa. As potential biomarkers, 113 bacterial genera were identified in the basal stem tissues, among which, the top 5 genera with the highest LDA scores were *Bacillus*, *Kosakonia*, *Pseudomonas*, *Acinetobacter*, and *Parapedobacter* (Table S3). Moreover, 78 bacterial biomarkers were screened from the root-zone soil; the top 5 were *Tumebacillus*, *Effusibacillus*, *Brevibacillus*, *Cohnella*, and *Alicyclobacillus* (Table S4).

In terms of fungal communities, 48 fungal genera were confirmed as biomarkers in basal stem tissues, with the top 6 genera being *Fusarium*, *Mucor*, *Acremonium*, *Phaeophleospora*, *Paraconiothyrium*, and *Penicillium* (Table S5). A total of 51 fungal biomarkers, including *Apiotrichum*, *Acrocalymma*, *Lasiodiplodia*, *Preussia*, and *Neooccultibambusa*, in the root-zone soil (Table S6).

#### 3.3.4. Correlation Analysis Between Differential Fungi and Bacteria

**Figure 6:**
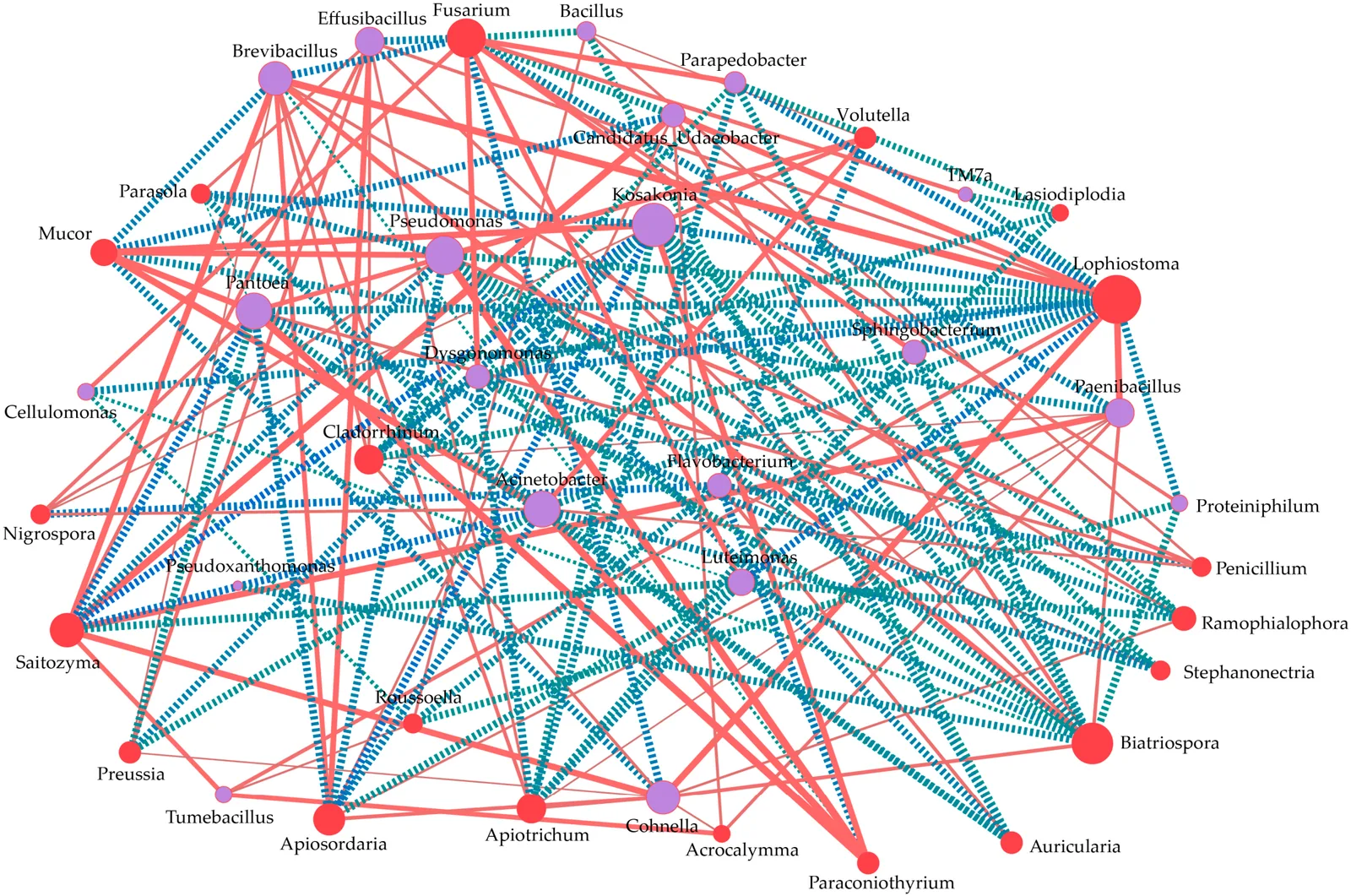
Correlation network among the top 20 most abundant fungal and bacterial genera, inferred using the SparCC algorithm. Red circular nodes represent fungal genera, and purple circular nodes represent bacterial genera; larger nodes indicate higher genus abundance. Only significant correlations with |r| > 0.5 and *p* < 0.05 are displayed. Solid lines denote positive correlations, dashed lines denote negative correlations, and line thickness is proportional to the absolute value of the correlation coefficient.

To resolve the associations between fungal and bacterial genera, a co-occurrence network was constructed based on the SparCC algorithm using the top 20 dominant fungal and bacterial genera (Figure 6). Among the fungi, *Lophiostoma*, *Biatriospora*, and *Fusarium* showed the highest degree of connectivity with bacteria, whereas among the bacteria, *Pseudomonas*, *Pantoea*, *Kosakonia*, and *Acinetobacter* were most strongly linked to fungi. Notably, the pathogenic genus *Fusarium* showed significant negative correlations with beneficial bacterial taxa such as *Bacillus* and *Brevibacillus*. These negative associations suggest a potential ecological exclusion between pathogenic fungi and beneficial bacteria; however, the underlying antagonistic mechanisms remain to be experimentally validated.

### 3.4. Fungal Functional Guilds

**Figure 7:**
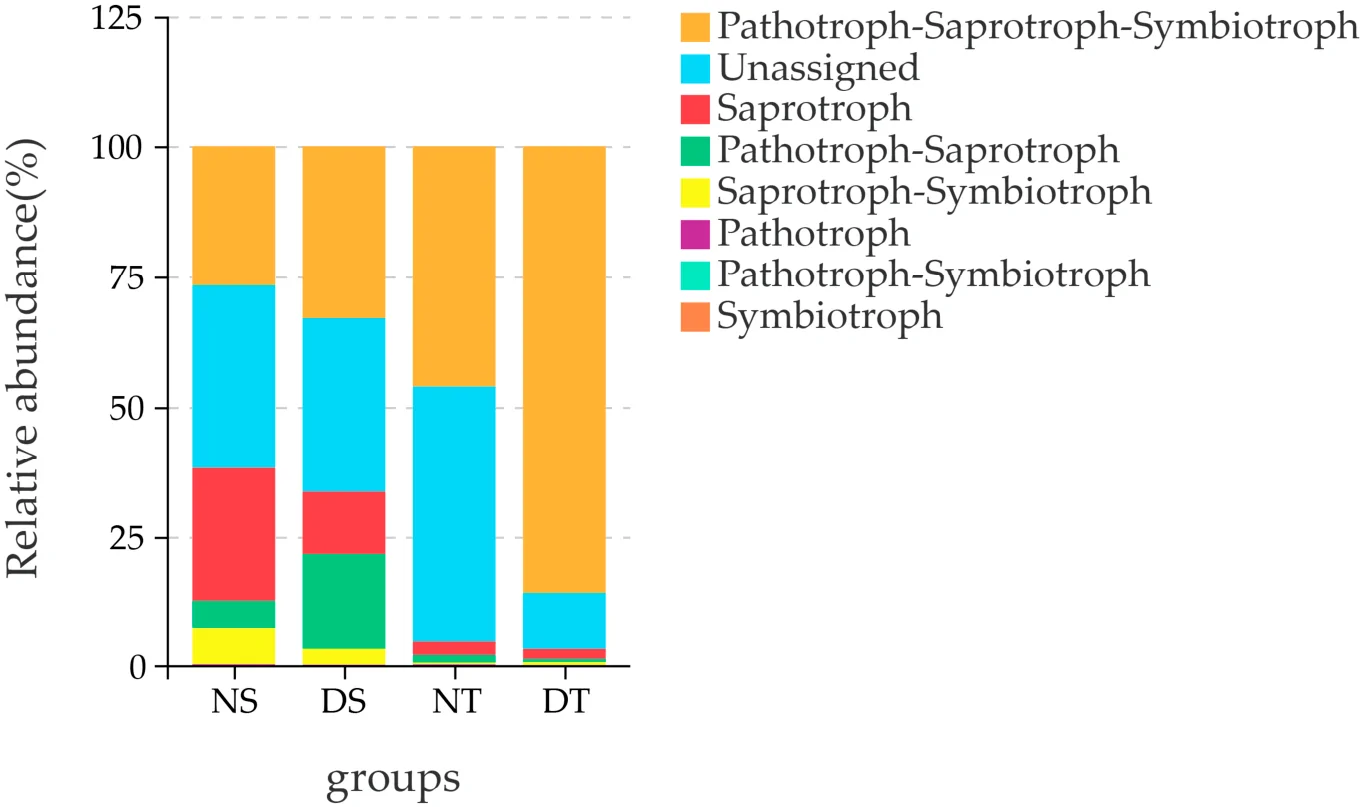
Distribution map of fungal trophic modes in root-zone soil and stem basal tissues of asymptomatic and diseased trees predicted based on the FUNGuild database. NS: Asymptomatic-tree root-zone soil; NT: Asymptomatic-tree basal stem tissue; DS: diseased-tree root-zone soil; DT: diseased-tree basal stem tissue. Trophic modes with relative abundances below 1% are not visually distinguishable in the main chart. Comprehensive data on all trophic modes are provided in Table S7.

FUNGuild functional prediction revealed that the trophic-mode composition of the fungal community differed markedly between asymptomatic and diseased samples. In the root-zone soil, asymptomatic samples (NS) were dominated by saprotrophs (Saprotroph, 25.59%) and Pathotroph–Saprotroph–Symbiotroph (26.69%), whereas diseased samples (DS) were dominated by Pathotroph–Saprotroph–Symbiotroph (32.98%) (Figure 7, Table S7). In basal stem tissue, 49.03% of sequences in asymptomatic samples (NT) remained unassigned to a defined trophic mode, whereas Pathotroph–Saprotroph–Symbiotroph fungi accounted for as much as 85.79% of the community in diseased samples (DT) (Figure 7, Table S7). These results indicate that, with foot rot development, the fungal community in diseased stem tissue becomes dominated by the Pathotroph–Saprotroph–Symbiotroph trophic mode.

### 3.5. Isolation, Identification, and Pathogenicity Verification of Fusarium Species

**Figure 8:**
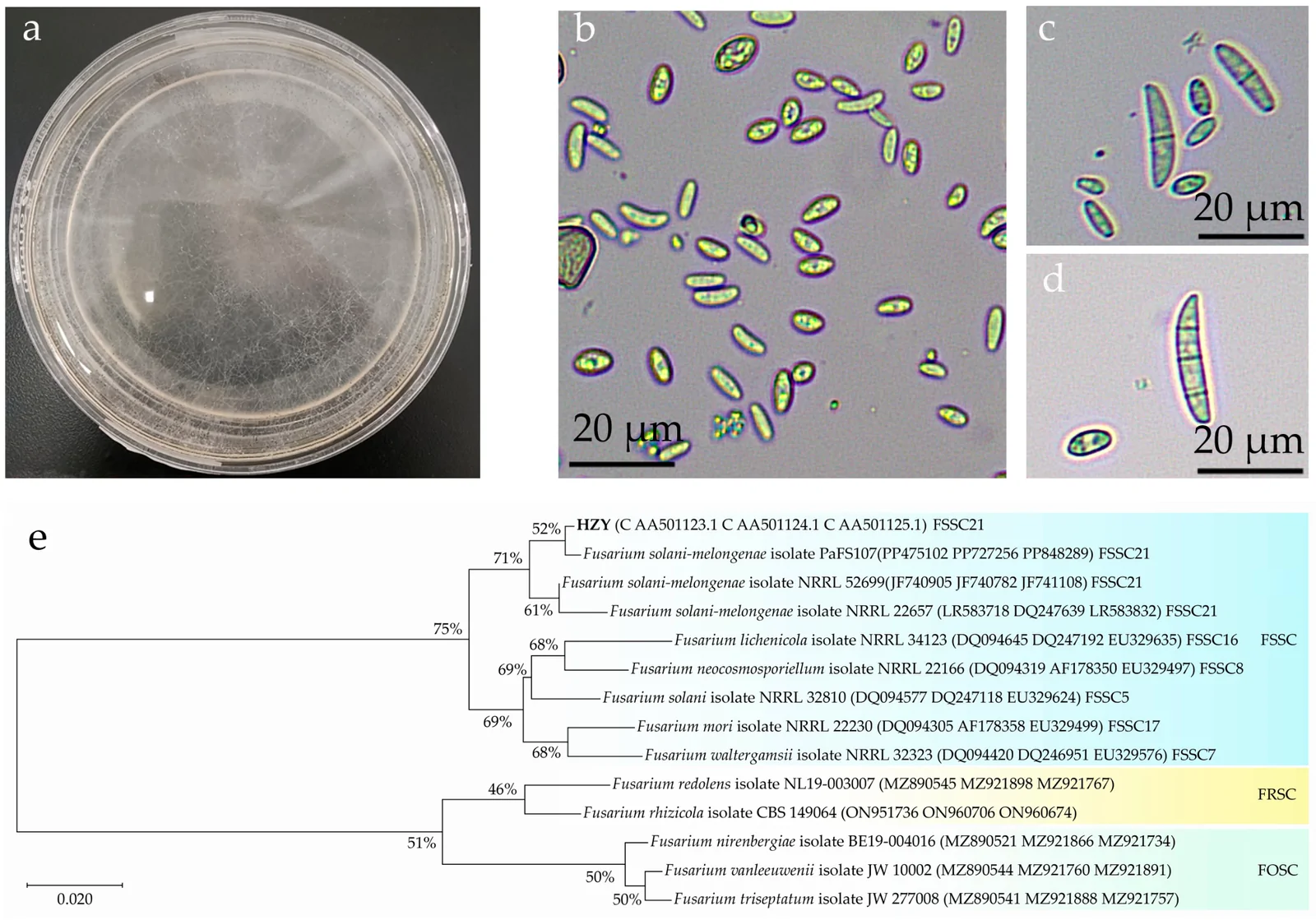
Colony morphology, microscopic characteristics, and multigene-based phylogenetic analysis of strain HZY. (**a**) Colony morphology; (**b**) conidia; (**c**,**d**) micro and macro septate conidia; (**e**) neighbor-joining phylogenetic tree constructed based on the concatenated sequences of ITS, EF-1α, and RPB2 genes. Phylogenetic trees were constructed in MEGA 12 using the neighbor-joining (NJ) method with the Kimura 2-parameter (K2P) model, pairwise deletion, and 1000 bootstrap replicates; the tree was rooted with *Fusarium oxysporum species complex* as the outgroup. Numbers at the nodes indicate bootstrap support values (%) based on 1000 replicates, and only values greater than 50% are shown; the scale bar indicates the number of nucleotide substitutions per site. HZY: the strain identified in this study; FSSC: *Fusarium solani* species complex; FRSC: *Fusarium redolens* species complex; FOSC: *Fusarium oxysporum* species complex.

Community analysis revealed that *Fusarium* abundance considerably increased in diseased tissues and root-zone soil, suggesting that *Fusarium* might be the causal pathogen of foot rot in *C. maxima* var. Tomentosa. Notably, *Fusarium*-like colonies with consistent morphological traits were isolated from all four diseased basal stem tissue samples; one representative isolate, designated HZY, was selected for further purification, multiloci molecular identification, and pathogenicity verification. This strain produced white, thin, and fluffy aerial mycelia and secreted yellow pigments (Figure 8a). Macroconidia were falciform, hyaline, and transparent with 2–5 septa, whereas microconidia were oval or reniform (Figure 8b–d).

Three gene fragments, *ITS* (539 bp), *EF-1α* (709 bp), and *RPB2* (943 bp), were amplified using the primer pairs ITS1/ITS4, EF-1/EF-2, and fRPB2-5f2/fRPB2-7cR, respectively (GenBank accession nos. CAAS0123.1, CAAS0124.1, and CAAS0125.1). BLAST (version 2.17.0) searches of the three sequences were conducted against the NCBI database. The results showed that the *ITS*, *EF-1α*, and *RPB2* sequences of the isolated strain shared 99.25%, 99.57%, and 100% sequence similarity with those of *F. solani-melongenae* isolate PaFS107, respectively. The neighbor-joining phylogenetic tree revealed that strain HZY clustered in the same terminal clade with *F. solani-melongenae* isolate PaFS107. This clade was further grouped with *F. solani-melongenae* isolate NRRL 22657 and *F. solani-melongenae* isolate NRRL 52699. These four strains together constitute the FSSC21 (*F. solani* species complex 21) lineage, forming a distinct branch parallel to other lineages within the FSSC, including FSSC16 (*F. lichenicola*), FSSC8 (*F. neocosmosporiellum*), FSSC5 (*F. solani*), FSSC17 (*F. mori*), and FSSC7 (*F. waltergamsii*) (Figure 8e). Meanwhile, HZY was phylogenetically distant from the FRSC and FOSC clades (Figure 8e). Taken together, the molecular phylogenetic results, combined with morphological characteristics, support the identification of strain HZY as *Fusarium solani-melongenae*, belonging to the FSSC21 lineage.

**Figure 9:**
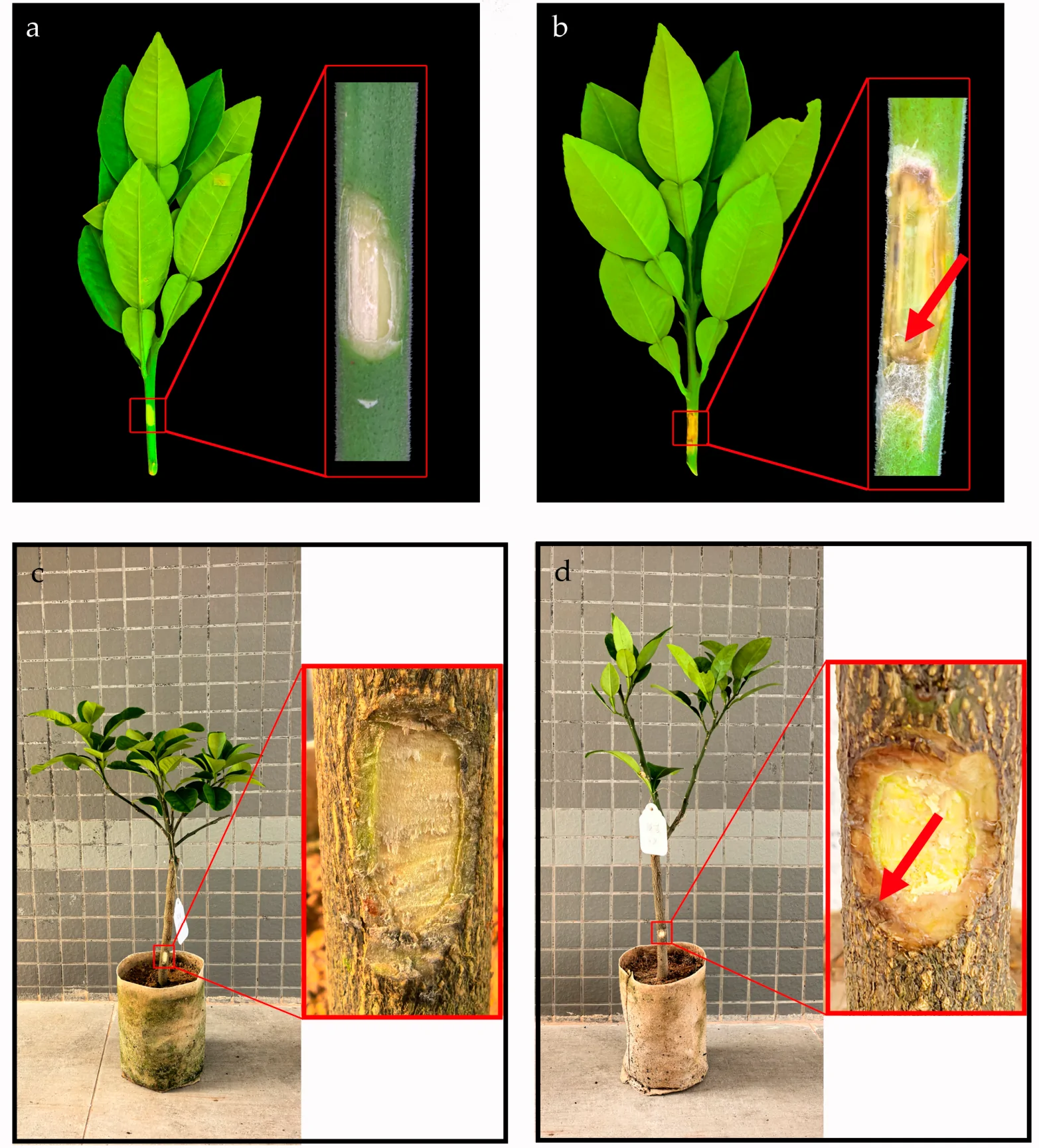
Disease symptoms of excised branches and stem bases of *Citrus maxima* var. Tomentosa following *Fusarium solani-melongenae* experimental inoculation. (**a**) Control branches treated with sterile water and enlarged view of inoculation sites; (**b**) pathogen-inoculated branches and enlarged view of inoculation sites; (**c**) control trees treated with sterile water and enlarged view of inoculation sites; (**d**) pathogen-inoculated trees and enlarged view of inoculation sites. Red arrows indicate diseased tissues with yellowed and decayed cortex. Three plants and three detached branches were used for each treatment.

To verify the pathogenicity of the isolated strain, experimental inoculation assays were performed on healthy *C. maxima* var. Tomentosa trees. No obvious necrotic or rotting lesions were observed on the bark of branches and stem bases in the control group inoculated with sterile agar plugs (Figure 9a,c). In contrast, typical foot-rot symptoms developed on branches and stem bases after inoculation with *Fusarium solani-melongenae* (Figure 9b,d). Cortical yellowing and tissue decay occurred at the inoculation sites indicated by red arrows. The symptoms reproduced by experimental inoculation were consistent with those observed in naturally infected field plants. The pathogen was re-isolated from the diseased tissues of symptomatic shoots and stem bases. The re-isolates exhibited completely identical morphological features to the originally inoculated *F. solani-melongenae* (Figure S1A). Sequence alignment of *ITS*, *EF-1α*, and *RPB2* showed 100% sequence identity between the re-isolates and original inoculum (Figure S1B–D). Collectively, these results complied with Koch’s postulates, confirming that *F. solani-melongenae* is the causal agent identified under the tested conditions of foot rot disease in *C. maxima* var. Tomentosa in the surveyed plot.

## 4. Discussion

Foot rot of *C. maxima* var. Tomentosa was closely associated with microecological dysbiosis in the root-zone soil and basal stem tissues, characterized by reduced microbial diversity, the collapse of putatively beneficial taxa such as *Bacillus*, and the pronounced enrichment of *Fusarium*. Through community profiling combined with pathogen isolation and verification of Koch’s postulates, *F. solani-melongenae* was identified as a causal agent under the tested conditions in the surveyed plot. These findings provide a reference for integrated, microecology-based disease management—warranting further research into the etiology, epidemiology, and ecological drivers of this disease.

The richness and variability of soil microbial communities affect soil quality, ecosystem function, sustainability, and plant health [42]. A higher alpha diversity indicates a more complex and stable microbial community structure in specific habitats [43]. In the present study, foot rot significantly reduced bacterial and fungal diversity in the root-zone soil. However, in diseased basal stem tissues, bacterial diversity increased whereas fungal diversity decreased, suggesting that the disease disrupted the complexity and stability of local microhabitats [44]. As a key habitat involved in disease development, the root-zone soil often exhibits reduced microbial diversity, a pattern widely associated with pathogen accumulation in soil-borne disease studies of *Atractylodes lancea* (Thunb.) DC. [45], cabbage [46], and grapes [47]. In the present study, in diseased basal stem tissues, bacterial community restructuring was associated with increased diversity, whereas pathogenic fungi dominated fungal communities and thus exhibited decreased diversity. The increased bacterial diversity in diseased basal stem tissues may reflect the colonization of pathogen-created niches: tissue necrosis releases nutrients and disrupts physical barriers, facilitating the establishment of diverse saprophytic and opportunistic bacteria. In addition, pathogen-induced plant defense responses may alter the tissue microenvironment, selectively allowing a broader array of bacterial taxa to survive. Nevertheless, because necrotic lesions and intact epidermis represent different tissue states, this diversity increase should be interpreted with caution and may partly reflect secondary colonization of decaying tissue. These results are consistent with previous findings on endophytic microbial diversity in root-rot-diseased *Panax notoginseng* (Burkill) F.H.Chen and *Angelica sinensis* (Oliv.) Diels [48,49]. PCoA further confirmed the significant separation of microbial communities between asymptomatic and diseased samples, indicating that foot rot infection substantially reshaped the microbial assembly in the root-zone and basal stem tissues of *C. maxima* var. Tomentosa [49].

In terms of community composition, asymptomatic basal stem tissues were enriched in beneficial genera including *Bacillus*, *Kosakonia*, *Pseudomonas*, and *Acinetobacter*. *Bacillus* exhibits broad-spectrum antimicrobial activity and is widely used as a biological control agent against various crop diseases, including foot rot [50,51]. *Kosakonia* serves as a plant growth-promoting bacterium, improving crop yield and quality [52,53]. *Pseudomonas* exerts biocontrol effects via nutrient and niche competition and effectively inhibits foot rot pathogens [54,55]. *Acinetobacter* enhances plant stress tolerance and promotes plant growth [56,57]. The abundance of these beneficial microbes sharply decreased in diseased tissues in the present study, suggesting a collapse of the original beneficial microecological balance. Moreover, the fungal community structure was severely disturbed, with the relative abundance of pathogenic *Fusarium* reaching 62.97%. *Bacillus*, *Paenibacillus*, and *Tumebacillus* were consistently the dominant bacterial genera in both asymptomatic and diseased root-zone soils, whereas fungal communities underwent more drastic shifts. The relative abundances of *Fusarium* and *Pyrenochaeta*, two well-known phytopathogenic fungi, were significantly elevated in root-zone soils of diseased plants [58,59]. The LEfSe analysis in the present study further verified that *Fusarium* was the core differential genus, and SparCC analysis revealed a strong negative correlation between *Fusarium* and *Bacillus*. *Bacillus* strains can effectively antagonize *Fusarium* pathogens and serve as ideal biocontrol resources for *Fusarium*-induced diseases [50,51]. Combined with previous reports on the antifungal activity of *Bacillus* against *Fusarium*, the negative association observed in the present study suggests that the decreased abundance of beneficial *Bacillus* may facilitate the colonization and proliferation of *Fusarium*. However, the actual antagonistic effect of these bacteria against the *Fusarium* strain in the present study requires further in vitro and in vivo validation. Fungal functional prediction confirmed a community functional transition: diseased root-zone soil had elevated proportions of pathotrophic and pathotroph-saprotrophic fungi, but decreased proportions of saprotrophic fungi, and the proportion of pathotroph-saprotroph-symbiotrophic fungi increased in diseased tissues. These results suggest a shift in the fungal community composition from saprotroph-dominated to pathotroph-dominated as the foot root disease progresses. However, these findings are based on taxonomic inference rather than direct functional measurements, warranting further confirmation using multi-omics methods, such as metagenomics or metatranscriptomics.

Although *Phytophthora* species are generally considered to be the primary causative pathogens of citrus foot rot [11,12,13,14,15], the high-throughput sequencing analysis in the present study detected no *Phytophthora* sequences in any of the tested samples. However, it should be noted that *Phytophthora* belongs to Stramenopila (Oomycota) rather than to Fungi, and its ITS region may be poorly amplified by the commonly used “fungal” ITS primers and may be underrepresented in the UNITE database. Consequently, the failure to detect *Phytophthora* in this study likely reflects a methodological limitation in primer and database coverage rather than a true biological absence. Likewise, pathogen isolation in the present study was performed only on PDA, a general-purpose medium that favors true fungi and does not selectively support oomycetes; no *Phytophthora*-specific baiting or isolation on oomycete-selective media was attempted. On the one hand, this fungus-biased isolation design means that the absence of *Phytophthora* among the isolates recovered on PDA cannot be taken as evidence of its absence in the orchard, and a potential oomycete contribution may have been overlooked. On the other hand, it is exactly this fungal-biased isolation framework that caused the readily recoverable agent from diseased tissues to be a true fungus rather than an oomycete. Therefore, it cannot be rule out the possibility that *Phytophthora* may act as a co-pathogen in the disease complex. Against this background, the proliferation of *Fusarium* was found to be closely associated with the occurrence of foot rot in the present study. A fungal strain was isolated from diseased tissues of *C. maxima* var. Tomentosa and identified as *F. solani-melongenae*, a typical soil-borne phytopathogen of the *F. solani* species complex (phylogenetic clade FSSC 21) [60]. This species infects a wide range of host plants, including sweet potato, passion fruit, and coffee [60,61,62]. The pathogen invades plants mainly through wounds or natural openings, causing vascular browning and epidermal decay. In severe cases, leaves wilt and abscise, eventually leading to whole-plant wilting and death [61,63]. The back-inoculation experiments on the shoots and basal stems of *C. maxima* var. Tomentosa fulfilled Koch’s postulates for isolate HZY, confirming its pathogenicity under the tested conditions. Collectively, these results support *F. solani-melongenae* as a causal agent of foot rot in the surveyed plot. While this finding suggests that *F. solani-melongenae* may be involved in the pathogenesis of foot rot in this cultivar, the present sequencing design does not permit the exclusion of oomycetes. Thus, the role of *F. solani-melongenae* as a pathogen should be interpreted with caution, and future studies employing oomycete-selective media, *Phytophthora*-specific primers, or qPCR assays are warranted to clarify the relative contributions of oomycetes and true fungi in this disease complex.

The occurrence of foot rot in *C. maxima* var. Tomentosa was closely associated with microecological dysbiosis in the root-zone soil and basal stem tissues. The enrichment of *Fusarium* fungi in these niches, especially the marked enrichment of *F. solani-melongenae*, may contribute to foot rot development; however, it should be regarded as one of several possible triggers because disease onset is also governed by predisposing factors, including bioclimatic conditions, orchard management, fertilization, excessive irrigation, and planting density. Nevertheless, some limitations of this study should be acknowledged. First, the samples were only collected from a single orchard; consequently, the findings cannot be generalized to other orchards, as variable environmental conditions across different planting sites may shape distinct plant and soil microbial communities. Second, the relatively small sample size may have reduced the statistical robustness of the microbiome analysis and weakened the statistical power to detect low-abundance differentially abundant taxa. Third, the physicochemical properties of root-zone soil were not determined. Although all samples were collected from the same orchard under uniform agronomic management, microscale soil heterogeneity may exist and may have confounded the interpretation of disease status effects on microbial community composition. Fourth, the pathogenicity assay was preliminary, being based on a single isolate (HZY) and three biological replicates, with experimental wound inoculation of one-year-old seedlings and excised branches and without quantification or statistical analysis of disease severity. The results thus provide initial support of Koch’s postulates rather than a quantitative comparison of aggressiveness; field validation with multiple isolates and larger host populations is warranted. Future studies should aim to investigate the dynamic succession of microbial communities and more accurately determine the etiology and epidemiology of the disease across different disease progression stages and multiple orchards and analyze soil physicochemical properties to clarify the key factors that drive the evolution of microbial community structure. Screening highly efficient beneficial antagonistic microorganisms and conducting microecological regulation trials will provide technical support for constructing a green and sustainable control system for foot rot disease of *C. maxima* var. Tomentosa.

## 5. Conclusions

In this study, foot rot disease was associated with reduced microbial diversity in the root-zone soil and with distinct restructuring of the microbial communities in both root-zone soil and basal stem niches. Specifically, the relative abundance of potentially beneficial genera (e.g., *Bacillus*) was significantly lower in diseased samples, whereas the relative abundance of pathogenic fungi represented by *Fusarium* was markedly increased. FUNGuild-based prediction revealed that the fungal community shifted from a saprotroph-dominated pattern to a pathotroph-enriched pattern with disease progression. These results reflect community changes associated with foot rot disease. In this investigation, *F. solani-melongenae* was confirmed as the causal agent of foot rot disease in *C. maxima* var. Tomentosa. Overall, these findings provide preliminary insights into the microecological patterns associated with foot rot disease and offer a reference for developing green disease management strategies through root-zone microecological regulation and beneficial microorganism application.

## Figures and Tables

**Table 1:** Permutational multivariate analysis of variance (PERMANOVA; Adonis) test of microbial communities in basal stem tissues and root-zone soil of *Citrus maxima* Tomentosa.

Group Comparison	Bacterial			Fungal		
	F	R^2^	*p*	F	R^2^	*p*
NS-vs.-DS	4.3241	0.4188	0.031	3.0485	0.3369	0.024
NT-vs.-DT	14.1298	0.7019	0.033	8.4646	0.5852	0.020

**Table 2:** Dispersion test of microbial communities in basal stem tissues and root-zone soil of *Citrus maxima* Tomentosa.

Group Comparison	Sample ID	Bacterial			Fungal		
		Average Dispersion	F	*p*	Average Dispersion	F	*p*
NS-vs.-DS	NS	0.1885	0.0115	0.978	0.3565	8.0068	0.057
	DS	0.1908			0.2821		
NT-vs.-DT	NT	0.2071	26.266	0.030	0.349	14.1444	0.028
	DT	0.3266			0.1672		

## Data Availability

The raw sequence data reported in this paper have been deposited at the National Genomics Data Center (https://ngdc.cncb.ac.cn/, accessed on 14 May 2026) under the accession number PRJCA064203. The *ITS*, *EF-1α*, and *RPB2* sequences of the HZY isolate have been deposited in the National Genomics Data Center (https://ngdc.cncb.ac.cn/genbase, accessed on 7 September 2026) under accession numbers C_AA501123.1, C_AA501124.1, and C_AA501125.1, respectively. The data are presented in the article and Supplementary Materials.

## Supplementary Materials

The following supporting information can be downloaded at https://www.mdpi.com/article/10.3390/microorganisms14102258/s1. Figure S1: Morphological and molecular identification of the pathogen re-isolated after back-inoculation; Table S1: Statistical table of Tags and OTUs numbers obtained by bacterial sequencing in root-zone soil and basal stem tissues of asymptomatic and diseased trees; Table S2: Statistical table of Tags and OTUs numbers obtained by fungal sequencing in root-zone soil and stem basal tissues of asymptomatic and diseased tree; Table S3: LDA statistical data of LEfSe analysis on bacteria communities in stem basal tissues of asymptomatic and diseased trees; Table S4: LDA statistical data of LEfSe analysis on bacteria communities in root-zone soil of asymptomatic and diseased trees; Table S5: LDA statistical data of LEfSe analysis on fungi communities in stem basal tissues of asymptomatic and diseased trees; Table S6: LDA statistical data of LEfSe analysis on fungi communities in root-zone soil of asymptomatic and diseased trees; and Table S7: Relative proportions of fungal trophic modes in rhizosphere soil and basal stem tissues of asymptomatic and diseased trees, as predicted by the FUNGuild database.
