# Supplementary material for: Differences in Root-Zone Soil and Basal Stem Tissue Microbial Communities Between Asymptomatic and Diseased Citrus maxima var. Tomentosa Plants with Foot Rot
Source: Microorganisms. 2026 Oct 4;14(10):2258. doi: 10.3390/microorganisms14102258 (PMC13707575; doi:10.3390/microorganisms14102258)
Supplement: Supplementary file 1 [file microorganisms-14-02258-s001.zip › Supplementary figure.pdf]

Supplementary figure

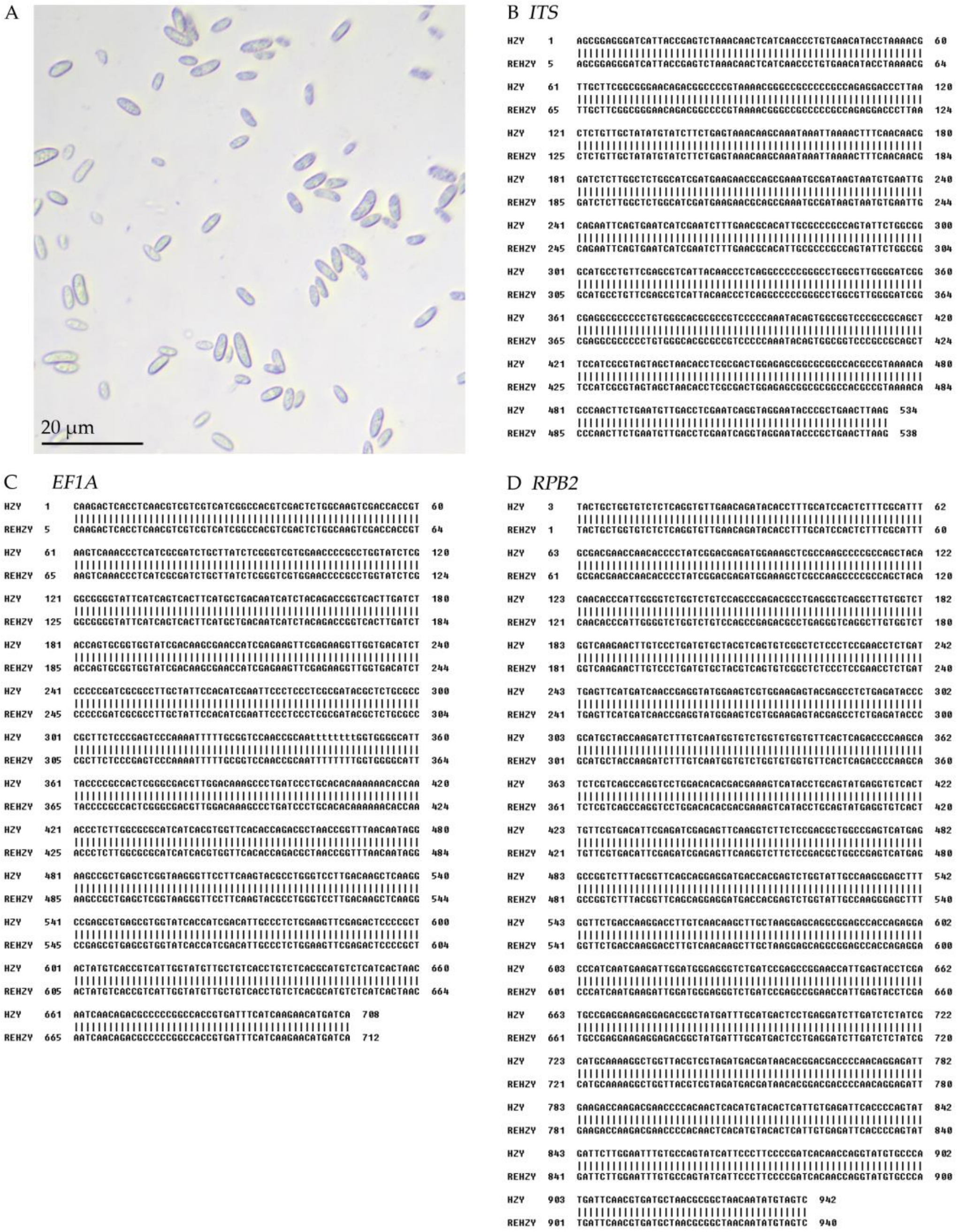

Figure S1 Morphological and molecular identification of the pathogen re-isolated after back-inoculation. A: Conidial morphology of the fungal strain; B: sequence alignment of the *ITS* gene between strain HZY and the re-isolated strain (REHZY) after back-inoculation; C: sequence alignment of the *EF-1α* gene between strain HZY and the re-isolated strain (REHZY) after back-inoculation; D: sequence alignment of the *RPB2* gene between strain HZY and the re-isolated strain (REHZY) after back-inoculation.
